# Supplementary material for: Peripheral Nervous System Adverse Events after the Administration of mRNA Vaccines: A Systematic Review and Meta-Analysis of Large-Scale Studies
Source: Vaccines (Basel). 2022 Dec 17;10(12):2174. doi: 10.3390/vaccines10122174 (PMC9781046; doi:10.3390/vaccines10122174)
Supplement: Supplementary file 1 [file vaccines-10-02174-s001.zip › vaccines-2082459-supplementary.pdf]

Study or Subgroup	Incidence Rate (per 100,000)	SE	Weight	Incidence Rate (per 100,000) IV, Random, 95% CI
**(a) Bell's palsy after mRNA (by case; first dose)**				
Li 2022 (AURUM; BNT162b2)	2.716343	0.400498	29.4%	2.72 [1.93, 3.50]
Li 2022 (SIDIAP; BNT162b2)	5.289791	0.528965	28.8%	5.29 [4.25, 6.33]
Li 2022 (SIDIAP; mRNA-1273)	5.726744	1.530493	21.4%	5.73 [2.73, 8.73]
Shasha 2021	9.864513	2.056792	17.4%	9.86 [5.83, 13.90]
McMurry 2021	27.02964	7.222998	3.0%	27.03 [12.87, 41.19]
**Subtotal (95% CI)**			**100.0%**	**6.07 [3.49, 8.65]**
Heterogeneity: Tau² = 5.72; Chi² = 35.72, df = 4 (P < 0.00001); I² = 89%				
Test for overall effect: Z = 4.62 (P < 0.00001)				
**(b) Bell's palsy after mRNA (by case; first dose; without study by McMurry)**				
Li 2022 (AURUM; BNT162b2)	2.716343	0.400498	31.8%	2.72 [1.93, 3.50]
Li 2022 (SIDIAP; BNT162b2)	5.289791	0.528965	31.0%	5.29 [4.25, 6.33]
Li 2022 (SIDIAP; mRNA-1273)	5.726744	1.530493	21.0%	5.73 [2.73, 8.73]
Shasha 2021	9.864513	2.056792	16.2%	9.86 [5.83, 13.90]
**Subtotal (95% CI)**			**100.0%**	**5.30 [3.03, 7.58]**
Heterogeneity: Tau² = 4.07; Chi² = 25.47, df = 3 (P < 0.0001); I² = 88%				
Test for overall effect: Z = 4.57 (P < 0.00001)				
**(c) Bell's palsy after mRNA (by case; second dose)**				
Li 2022 (SIDIAP; mRNA-1273)	2.057312	0.419943	27.7%	2.06 [1.23, 2.88]
Li 2022 (SIDIAP; BNT162b2)	3.125098	1.397564	23.1%	3.13 [0.39, 5.86]
Shasha 2021	6.105332	2.158495	18.5%	6.11 [1.87, 10.34]
Li 2022 (AURUM; BNT162b2)	6.790873	0.736549	26.6%	6.79 [5.35, 8.23]
McMurry 2021	20.48236	7.240866	4.1%	20.48 [6.29, 34.67]
**Subtotal (95% CI)**			**100.0%**	**5.06 [1.97, 8.16]**
Heterogeneity: Tau² = 8.82; Chi² = 38.55, df = 4 (P < 0.00001); I² = 90%				
Test for overall effect: Z = 3.21 (P = 0.001)				
**(d) Bell's palsy after mRNA (by case; second dose; without study by McMurry)**				
Li 2022 (10)	2.057312	0.419943	29.3%	2.06 [1.23, 2.88]
Li 2022 (11)	3.125098	1.397564	23.9%	3.13 [0.39, 5.86]
Shasha 2021	6.105332	2.158495	18.7%	6.11 [1.87, 10.34]
Li 2022 (12)	6.790873	0.736549	28.0%	6.79 [5.35, 8.23]
**Subtotal (95% CI)**			**100.0%**	**4.40 [1.42, 7.38]**
Heterogeneity: Tau² = 7.70; Chi² = 32.92, df = 3 (P < 0.00001); I² = 91%				
Test for overall effect: Z = 2.89 (P = 0.004)				
**(e) Bell's palsy after mRNA (by dose)**				
Barda 2021	8.769157	0.974308	17.9%	8.77 [6.86, 10.68]
Bardenheier 2021	12.08459	12.08386	16.0%	12.08 [-11.60, 35.77]
Polack 2021	21.27546	10.6366	16.4%	21.28 [0.43, 42.12]
Walker 2022 (mRNA-1273)	30.53483	3.456861	17.7%	30.53 [23.76, 37.31]
El Sahly 2021	52.68704	18.62277	14.1%	52.69 [16.19, 89.19]
Walker 2022 (BNT162b2)	62.99362	1.048253	17.9%	62.99 [60.94, 65.05]
**Subtotal (95% CI)**			**100.0%**	**31.08 [1.44, 60.71]**
Heterogeneity: Tau² = 1278.40; Chi² = 1442.13, df = 5 (P < 0.00001); I² = 100%				
Test for overall effect: Z = 2.06 (P = 0.04)				

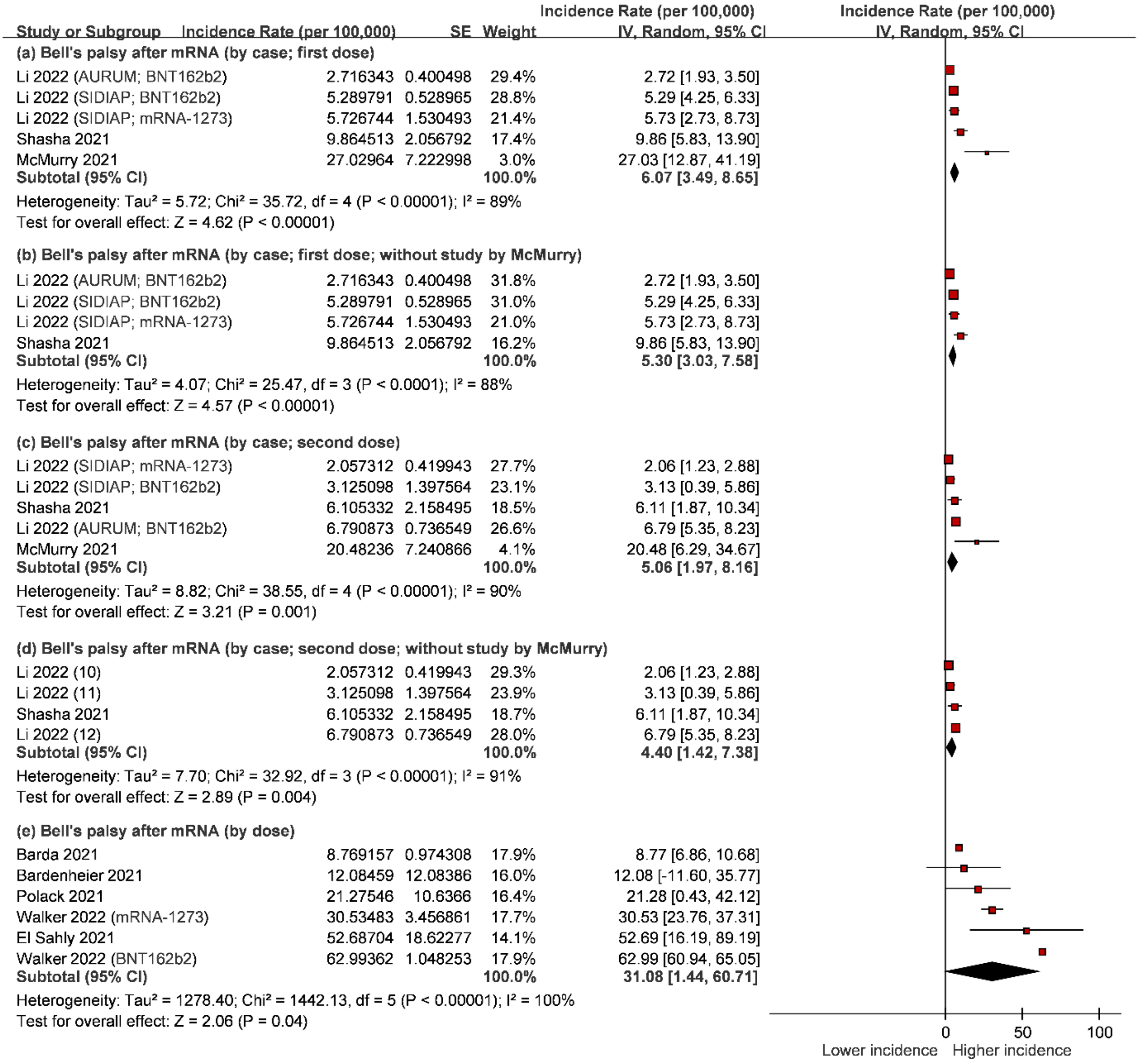

Figure S1. Pooled analysis of the incidence of Bell's palsy as an adverse event of mRNA vaccines against coronavirus disease 2019.

Study or Subgroup	mRNA vaccination Events	Total	Unvaccinated Events	Total	Weight	Peto Odds Ratio Peto, Fixed, 95% CI
**Bell's palsy, mRNA vaccination vs Unvaccinated**						
McMurry 2021	26	68266	75	68266	33.2%	0.38 [0.26, 0.56]
Shasha 2021	23	233159	24	233159	15.5%	0.96 [0.54, 1.70]
Barda 2021	81	923692	59	923692	46.1%	1.37 [0.98, 1.91]
El Sahly 2021	8	15184	3	15162	3.6%	2.48 [0.76, 8.09]
Polack 2021	4	18801	0	18785	1.3%	7.38 [1.04, 52.42]
Bardenheier 2021	1	8275	0	11072	0.3%	10.36 [0.20, 544.33]
**Total (95% CI)**		**1267377**		**1270136**	**100.0%**	**0.89 [0.71, 1.11]**
Total events	143		161			

Heterogeneity: Chi² = 33.82, df = 5 (P < 0.00001); I² = 85%
Test for overall effect: Z = 1.02 (P = 0.31)

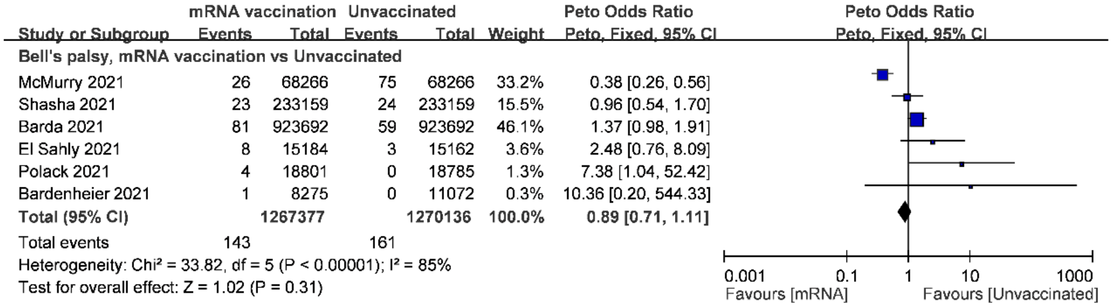

Figure S2. Forest plot analysis of the incidence of Bell's palsy in the mRNA vaccination and control groups including McMurry et al.'s study.

Study or Subgroup	mRNA vaccination Events	mRNA vaccination Total	Unvaccinated Events	Unvaccinated Total	Weight	Peto Odds Ratio Peto, Fixed, 95% CI
**(a) Bell's palsy (BNT162b2, First dose)**						
McMurry 2021	14	51795	35	51795	50.0%	0.42 [0.24, 0.74]
Shasha 2021	23	233159	24	233159	48.0%	0.96 [0.54, 1.70]
Polack 2021	1	18801	0	18785	1.0%	7.38 [0.15, 372.07]
Bardenheier 2021	1	5842	0	11072	0.9%	18.09 [0.29, 1115.63]
**Subtotal (95% CI)**		**309597**		**314811**	**100.0%**	**0.67 [0.45, 0.99]**
Total events	39		59			
Heterogeneity: Chi² = 7.96, df = 3 (P = 0.05); I² = 62%						
Test for overall effect: Z = 1.99 (P = 0.05)						
**(b) Bell's palsy (BNT162b2, Second dose)**						
Bardenheier 2021	0	5842	0	11072		Not estimable
McMurry 2021	8	39058	20	39058	54.9%	0.42 [0.20, 0.89]
Shasha 2021	8	131033	12	131033	39.2%	0.67 [0.28, 1.61]
Polack 2021	3	18801	0	18785	5.9%	7.38 [0.77, 70.99]
**Subtotal (95% CI)**		**194734**		**199948**	**100.0%**	**0.60 [0.35, 1.04]**
Total events	19		32			
Heterogeneity: Chi² = 5.63, df = 2 (P = 0.06); I² = 64%						
Test for overall effect: Z = 1.82 (P = 0.07)						
**(c) Bell's palsy (BNT162b2, Mixed)**						
McMurry 2021	22	51795	55	51795	26.6%	0.42 [0.27, 0.66]
Shasha 2021	31	233159	36	233159	23.2%	0.86 [0.53, 1.39]
Barda 2021	81	923692	59	923692	48.5%	1.37 [0.98, 1.91]
Polack 2021	4	18801	0	18785	1.4%	7.38 [1.04, 52.42]
Bardenheier 2021	1	5842	0	11072	0.3%	18.09 [0.29, 1115.63]
**Subtotal (95% CI)**		**1233289**		**1238503**	**100.0%**	**0.93 [0.74, 1.17]**
Total events	139		150			
Heterogeneity: Chi² = 23.48, df = 4 (P = 0.0001); I² = 83%						
Test for overall effect: Z = 0.63 (P = 0.53)						

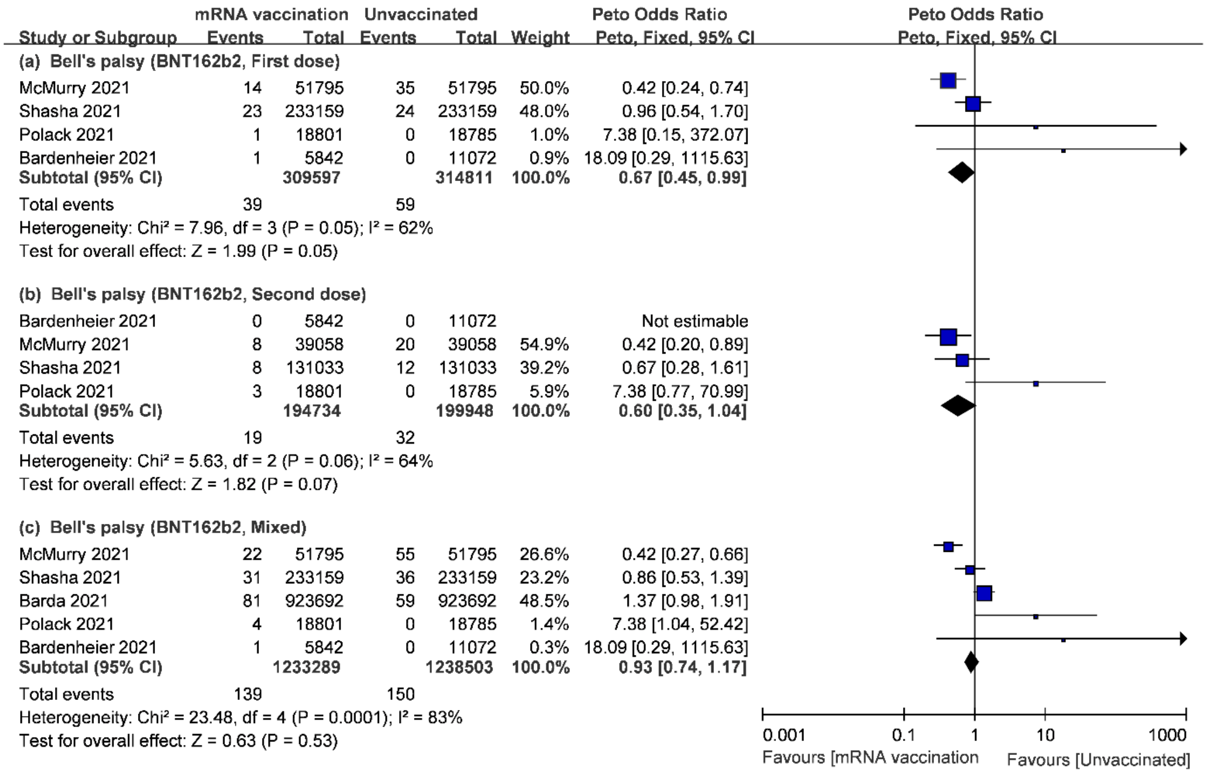

Figure S3. Forest plot analysis of the incidence of Bell's palsy after the first dose of BNT162b2 versus unvaccinated group (a), the second dose of BNT162b2 versus unvaccinated group (b), dose-unspecified BNT162b2 versus unvaccinated group (c), and BNT162b2 versus mRNA-1273 including McMurry et al.'s study.

Study or Subgroup	mRNA vaccination Events	mRNA vaccination Total	Unvaccinated Events	Unvaccinated Total	Weight	Risk Ratio M-H, Random, 95% CI	Risk Ratio M-H, Random, 95% CI
**(a) Bell's palsy, mRNA-1273 vs placebo (without study by McMurry et al.)**							
Bardenheier 2021	0	2433	0	11072		Not estimable	
El Sahly 2021	8	15184	3	15162	100.0%	2.66 [0.71, 10.04]	
**Subtotal (95% CI)**		**17617**		**26234**	**100.0%**	**2.66 [0.71, 10.04]**	
Total events	8		3				
Heterogeneity: Not applicable							
Test for overall effect: Z = 1.45 (P = 0.15)							
**(b) Bell's palsy, mRNA-1273 vs placebo**							
Bardenheier 2021	0	2433	0	11072		Not estimable	
McMurry 2021	4	16471	20	16471	51.2%	0.20 [0.07, 0.59]	
El Sahly 2021	8	15184	3	15162	48.8%	2.66 [0.71, 10.04]	
**Subtotal (95% CI)**		**34088**		**42705**	**100.0%**	**0.71 [0.06, 8.97]**	
Total events	12		23				
Heterogeneity: $Tau^2 = 2.98$; $Chi^2 = 8.86$, df = 1 (P = 0.003); $I^2 = 89\%$							
Test for overall effect: Z = 0.27 (P = 0.79)							

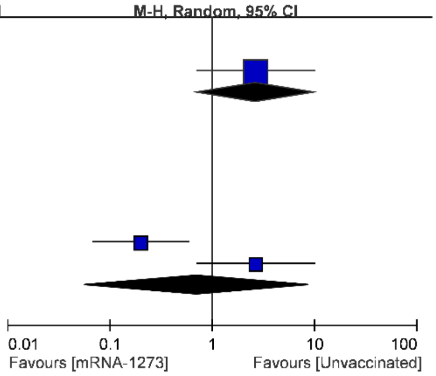

Figure S4. Forest plot analysis with (a) and without (b) McMurry et al.'s study of the incidence of Bell's palsy after the administration of mRNA-1273 SARS-CoV-2 vaccine compared with the control group.

Study or Subgroup	mRNA vaccination Events	mRNA vaccination Total	Unvaccinated Events	Unvaccinated Total	Weight	Peto Odds Ratio Peto, Fixed, 95% CI
**Bell's palsy (BNT162b2 vs mRNA-1273)**						
Li 2022	185	3142114	19	404462	11.6%	1.23 [0.80, 1.89]
McMurry 2021	22	51795	4	16471	2.7%	1.61 [0.66, 3.96]
Walker 2022	3609	5729152	78	255446	84.9%	1.69 [1.44, 1.99]
Koh 2021	11	1212130	0	185944	0.7%	3.17 [0.56, 18.06]
Bardenheier 2021	1	5842	0	2433	0.1%	4.12 [0.06, 304.42]
**Subtotal (95% CI)**		**10141033**		**864756**	**100.0%**	**1.64 [1.41, 1.90]**
Total events	3828		101			
Heterogeneity: Chi² = 2.59, df = 4 (P = 0.63); I² = 0%						
Test for overall effect: Z = 6.58 (P < 0.00001)						

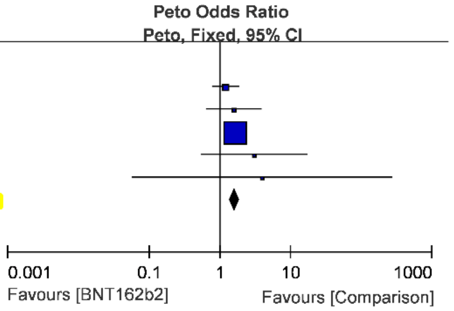

Figure S5. Forest plot analysis with (a) and without (b) McMurry et al.'s study on the incidence of Bell's palsy after BNT162b2 and mRNA-1273 vaccines.

Study or Subgroup	Incidence Rate (per 100,000)	SE	Weight	Incidence Rate (per 100,000) IV, Random, 95% CI
**(a) Guillain Barré syndrome after mRNA (by case)**				
Koh 2021 (BNT162b2)	0.164999	0.116672	6.6%	0.16 [-0.06, 0.39]
Keh 2022 (BNT162b2)	0.183	0.0321	87.4%	0.18 [0.12, 0.25]
Koh 2021 (mRNA-1273)	0.268898	0.380279	0.6%	0.27 [-0.48, 1.01]
Keh 2022 (mRNA-1273)	0.325	0.1367	4.8%	0.33 [0.06, 0.59]
Shasha 2021	0.428892	0.428891	0.5%	0.43 [-0.41, 1.27]
**Subtotal (95% CI)**			**100.0%**	**0.19 [0.13, 0.25]**
Heterogeneity: Tau² = 0.00; Chi² = 1.42, df = 4 (P = 0.84); I² = 0%				
Test for overall effect: Z = 6.34 (P < 0.00001)				
**(b) Guillain Barré syndrome after mRNA (by dose)**				
García- Grimshaw 2022 (mRNA-1273)	0.129419	0.07472	26.1%	0.13 [-0.02, 0.28]
García- Grimshaw 2022 (BNT162b2)	0.192231	0.033982	26.3%	0.19 [0.13, 0.26]
Walker 2022 (mRNA-1273)	0.195736	0.276812	23.9%	0.20 [-0.35, 0.74]
Walker 2022 (BNT162b2)	4.939649	0.293624	23.6%	4.94 [4.36, 5.52]
**Subtotal (95% CI)**			**100.0%**	**1.30 [0.42, 2.18]**
Heterogeneity: Tau² = 0.76; Chi² = 260.32, df = 3 (P < 0.00001); I² = 99%				
Test for overall effect: Z = 2.90 (P = 0.004)				

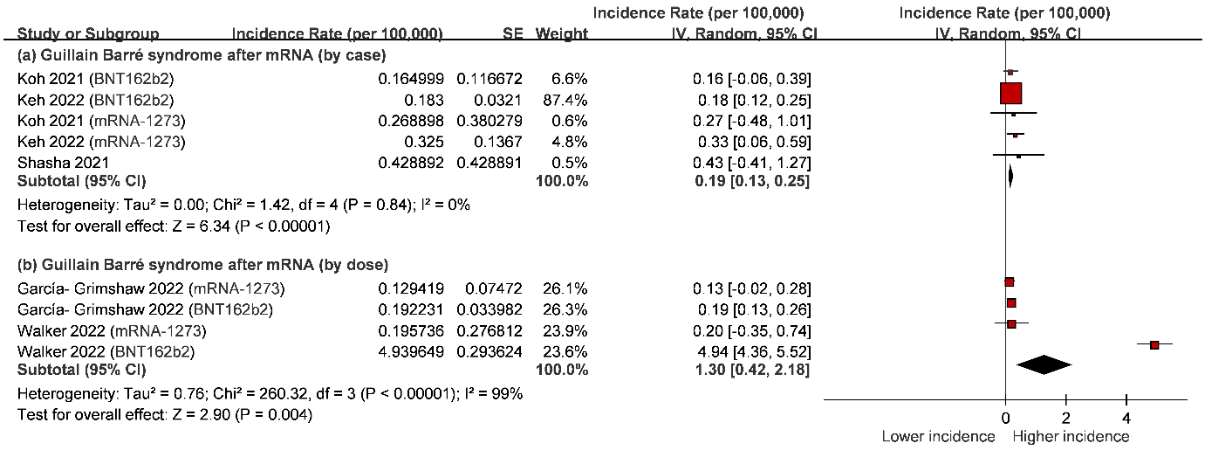

Figure S6. Forest plot analysis of the incidence of Guillain–Barré syndrome after the administration of mRNA vaccines.

Table S1. The Newcastle–Ottawa Scale (NOS) was used to evaluate the quality of the included cohort studies.

	Selection				Comparability		Outcome		
**Study**	**1**	**2**	**3**	**4**	**5a**	**5b**	**6**	**7**	**8**
**Barda et al.**	★	★	★	★	★	★	★	★	★
**Bardenheier et al.**		★	★	★	★	★	★		★
**Garcia-Grimshaw et al.**	★	★	★				★	★	★
**Hanson et al.**	★	★	★				★	★	★
**Keh et al.**	★	★	★				★	★	★
**Koh et al.**		★	★	★	★		★	★	★
**Li (Xintong) et al.**	★	★	★	★	★	★	★	★	★
**McMurry et al.**		★	★		★	★	★	★	★
**Renoud et al.**	★	★	★				★	★	★
**Rosenblum et al.**	★	★	★		★	★	★	★	★
**Sato et al.**	★	★	★				★	★	★
**Shasha et al.**	★	★	★	★	★	★	★		★
**Walker et al.**	★	★	★				★	★	★

1, Representativeness of the exposed cohort. 2, Selection of the non-exposed cohort. 3, Ascertainment of exposure. 4, Demonstration that the outcome of interest was not present at the start of the study. 5, Comparability of cohorts based on the design or analysis. 6, Assessment of outcome. 7, Was follow-up long enough for outcomes to occur? 8, Adequacy of follow-up of the cohorts.

The number of stars ranged from zero to nine (7–9 stars, high-quality; 4–6 stars, medium-quality; <4 stars, poor-quality).

Table S2. The Cochrane Risk-of-Bias Tool was used to evaluate the quality of the included randomized controlled trials.

**Trial**	**Item 1**	**Item 2**	**Item 3**	**Item 4**	**Item 5**	**Item 6**	**Item 7**
**NCT04470427 (Baden, El Sahly)**	Low	Low	Low	Low	Low	Low	High
**NCT04368728 (Polack, Stephen)**	Low	Low	Low	Low	Low	Low	High

1, Randomization; 2, Concealment. 3, Blinding (participants and professionals). 4, Blinding the assessor. 5, Lost to follow-up. 6, Selective reporting. 7, Other bias.

Each domain was categorized as "low," "high," or "unclear" risk of bias.
